# Supplementary material for: Structural basis of Fumosorinone-mediated allosteric inhibition of PTP1B for cancer immunotherapy
Source: Commun Biol. 2026 May 28;9:729. doi: 10.1038/s42003-026-10329-2 (PMC13219501; doi:10.1038/s42003-026-10329-2)
Supplement: Supplementary file 2 — supplementary information [file 42003_2026_10329_MOESM2_ESM.pdf]

Supplementary Information

This PDF file includes:

Supplementary Figures 1 to 11
Supplementary Table 1

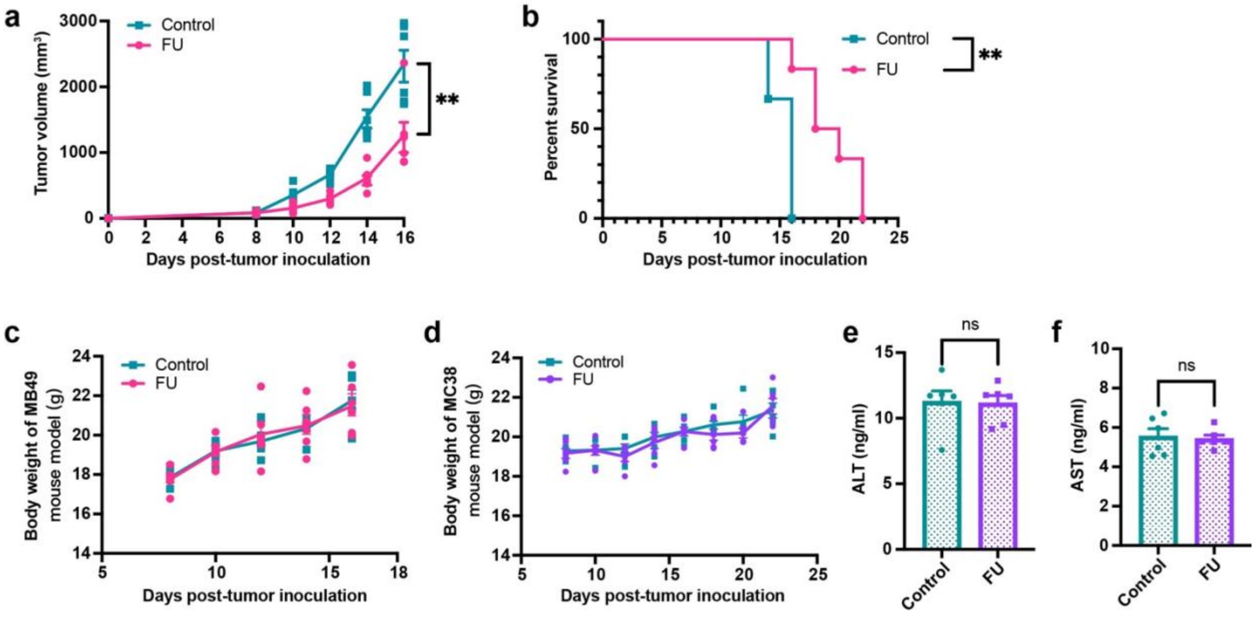

Supplementary Figure 1.

Antitumor efficacy and safety of FU in vivo. (a-c) Tumor growth curves (a), and survival analysis (b) in MB49-bearing mice treated with FU. (c-d) Body weight monitoring during FU treatment in MB49 (c) and MC38 (d) models. (e-f) Serum AST (e) and ALT (f) levels 24 hours post-FU administration (n = 6). Data are presented as mean ± SEM. Statistical significance: ns = not significant; *p < 0.05; **p < 0.01; ***p < 0.001; ****p < 0.0001.

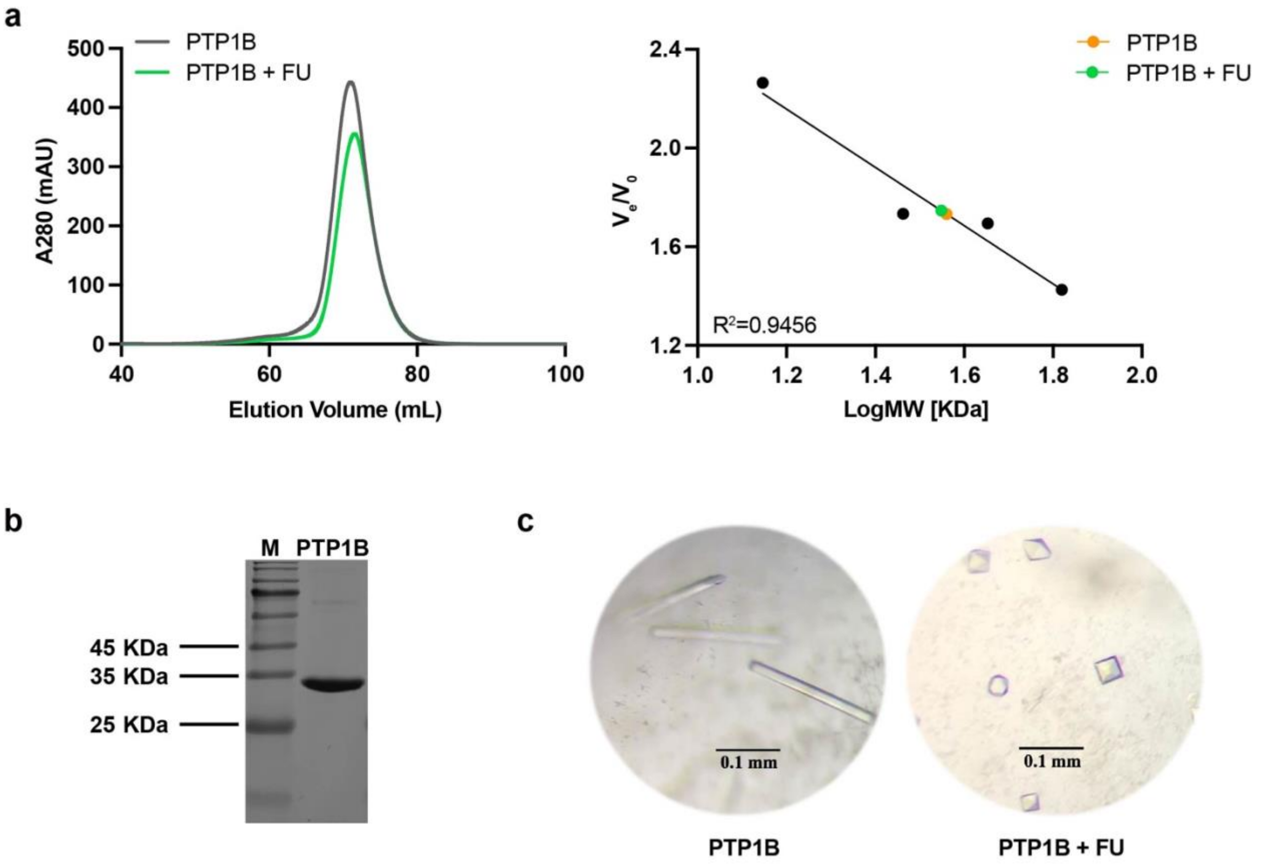

Supplementary Figure 2.

Purification and crystallization of PTP1B. (a) Size-exclusion chromatography on a Superdex 75 16/600 column shows that PTP1B remains monomer in solution with or without FU. (b) SDS-PAGE (15%) demonstrating purity of PTP1B. (c) Representative crystals of apo PTP1B and the PTP1B-FU complex.

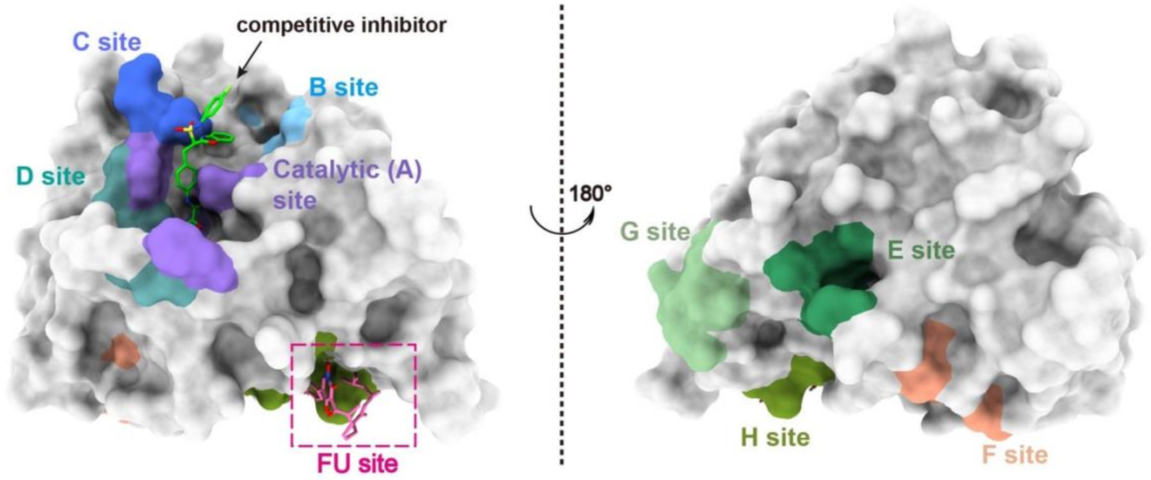

Supplementary Figure 3.

Structural mapping of PTP1B's allosteric and catalytic sites. Overview of binding pockets in the PTP1B-FU complex. Catalytic site (A site, purple) includes Tyr46, Gln262, Phe182, and Cys215. Secondary binding pockets (B-D) and distal allosteric sites (E-H) are labeled; the FU binding site (H site) is shown in hot pink. A representative competitive inhibitor bound to the catalytic pocket is shown in lime (PDB ID: 4I8N [48]).

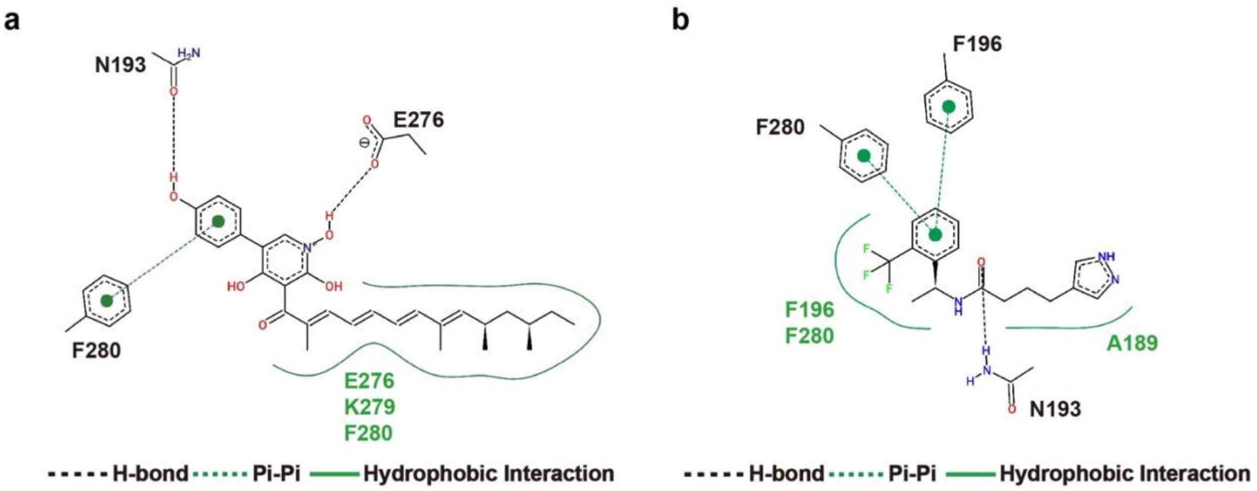

Supplementary Figure 4.

Interaction analysis of FU and PI-1 with PTP1B. (a-b) Molecular force interaction profiles for FU (a) and PI-1 (b) binding to PTP1B.

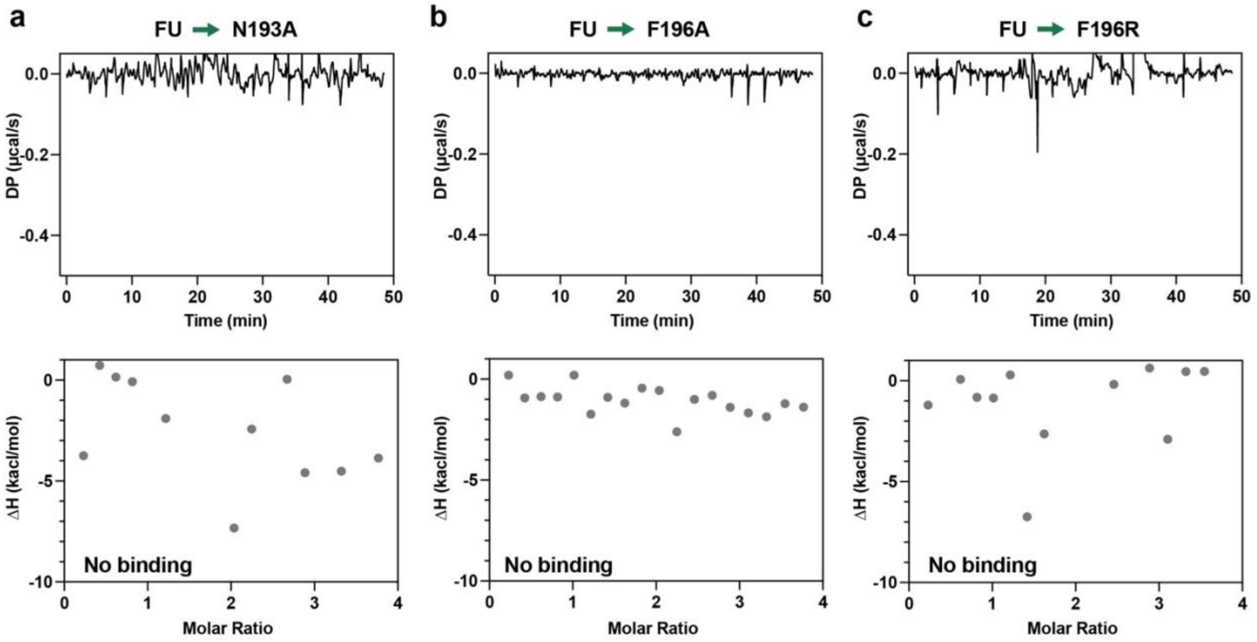

Supplementary Figure 5.

Interaction analysis of FU with PTP1B mutants. (a-c) ITC measurements showing that FU exhibits no detectable binding to the indicated PTP1B mutants, N193A (a), F196A (b), and F196R (c), under the experimental conditions used.

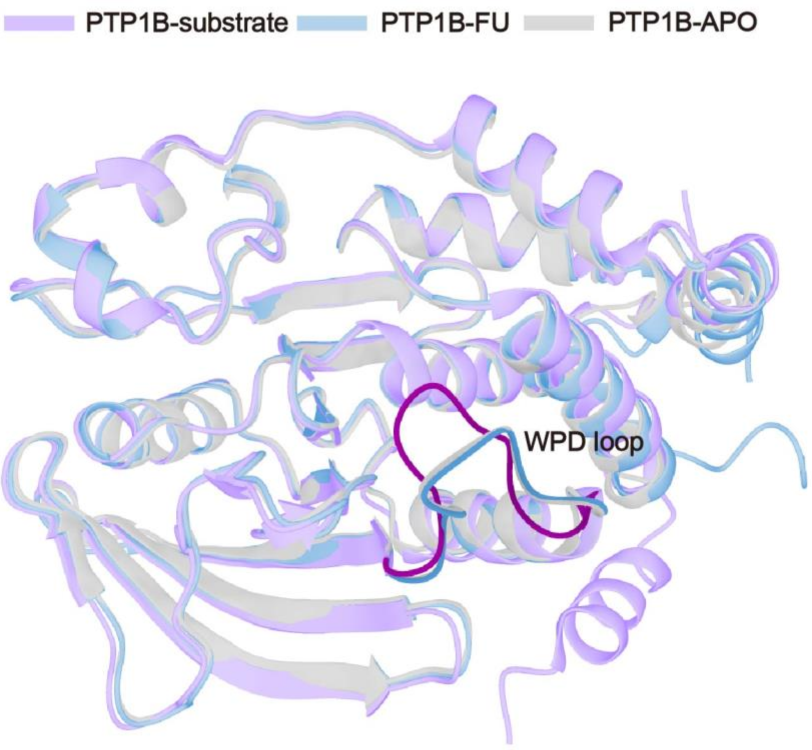

Supplementary Figure 6.

Structural comparison of PTP1B-FU, PTP1B-substrate, and PTP1B-APO. Superimposition of the PTP1B-FU complex (blue) and apo PTP1B-APO (grey) reveals minimal global deviation (RMSD = 0.45 Å), supporting the conclusion that FU stabilizes the open inactive conformation of PTP1B.

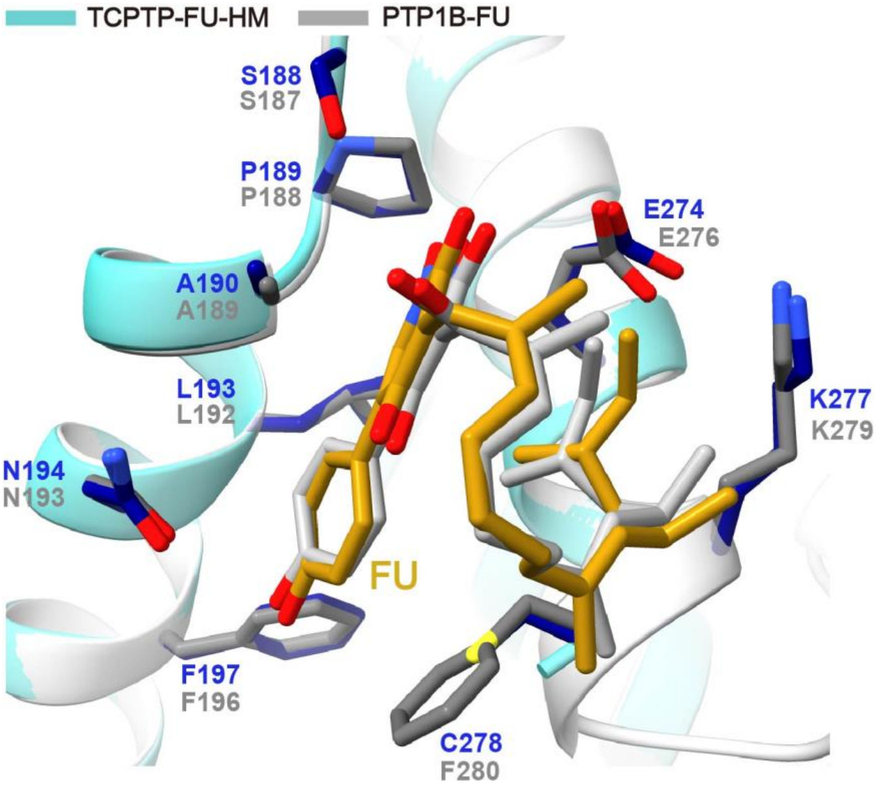

Supplementary Figure 7.

Comparison of FU binding modes in PTP1B and TCPTP. The TCPTP-FU complex structure was predicted by homology modeling based on the crystal structure of PTP1B-FU complex. The predicted model revealed a binding mode similar to that observed in PTP1B. FU interacts with the side chains of S188, P189, A190, L193, N194, F197, E274, K277, and C278 in TCPTP, corresponding to key residues involved in FU binding in PTP1B.

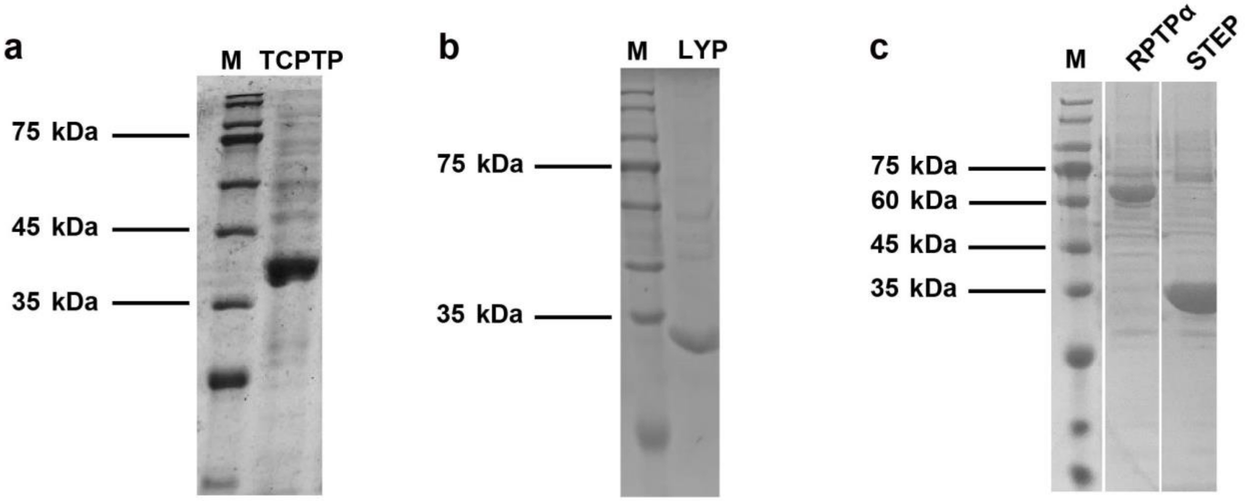

Supplementary Figure 8.

Purification of PTP1B-related phosphatases. SDS-PAGE (12%) of purified TCPTP, RPTPα, STEP and LYP, alongside molecular weight markers.

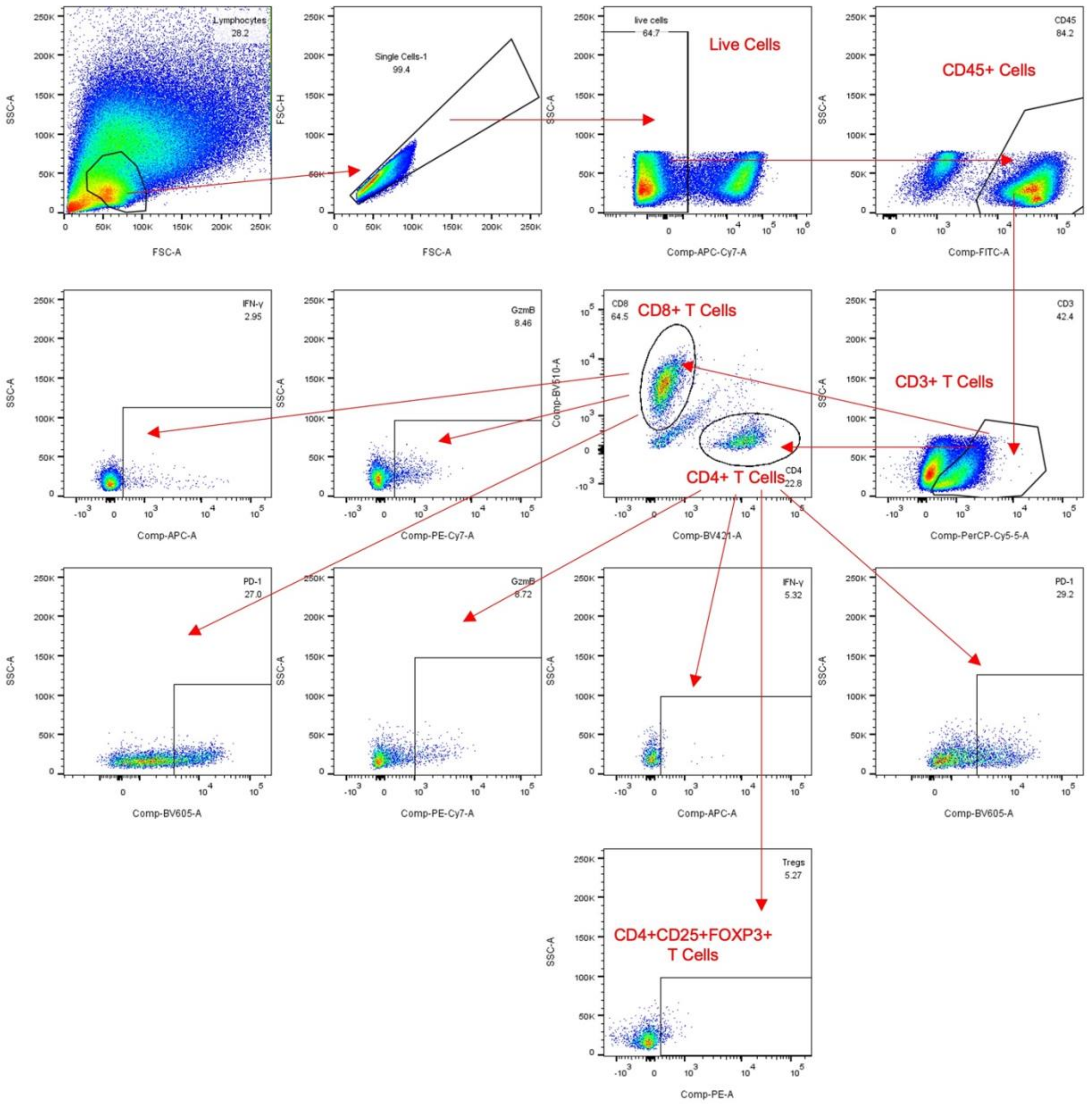

Supplementary Figure 9.

Gating strategy for flow cytometric analysis of T-cell subsets and effector functions. Cells isolated from tumor tissues were stained with fluorochrome-conjugated antibodies and analyzed by flow cytometry. Representative gating workflow illustrating the identification of T-cell subsets and their effector functional states.

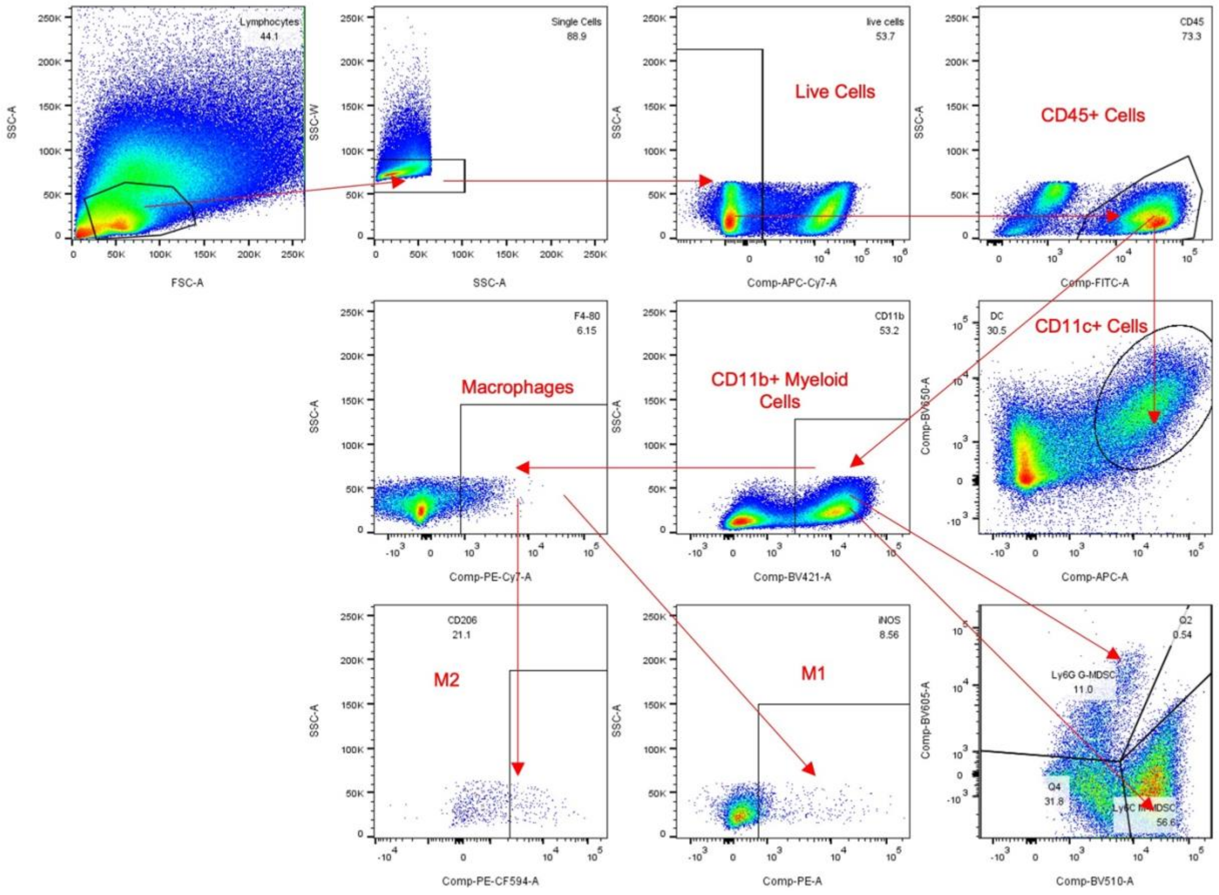

Supplementary Figure 10.

Gating strategy for flow-cytometric analysis of tumor microenvironment-associated immune cells. Representative gating workflow for the identification of tumor-infiltrating immune cell populations, including myeloid subsets, and the assessment of their functional states and relative abundances by flow cytometry.

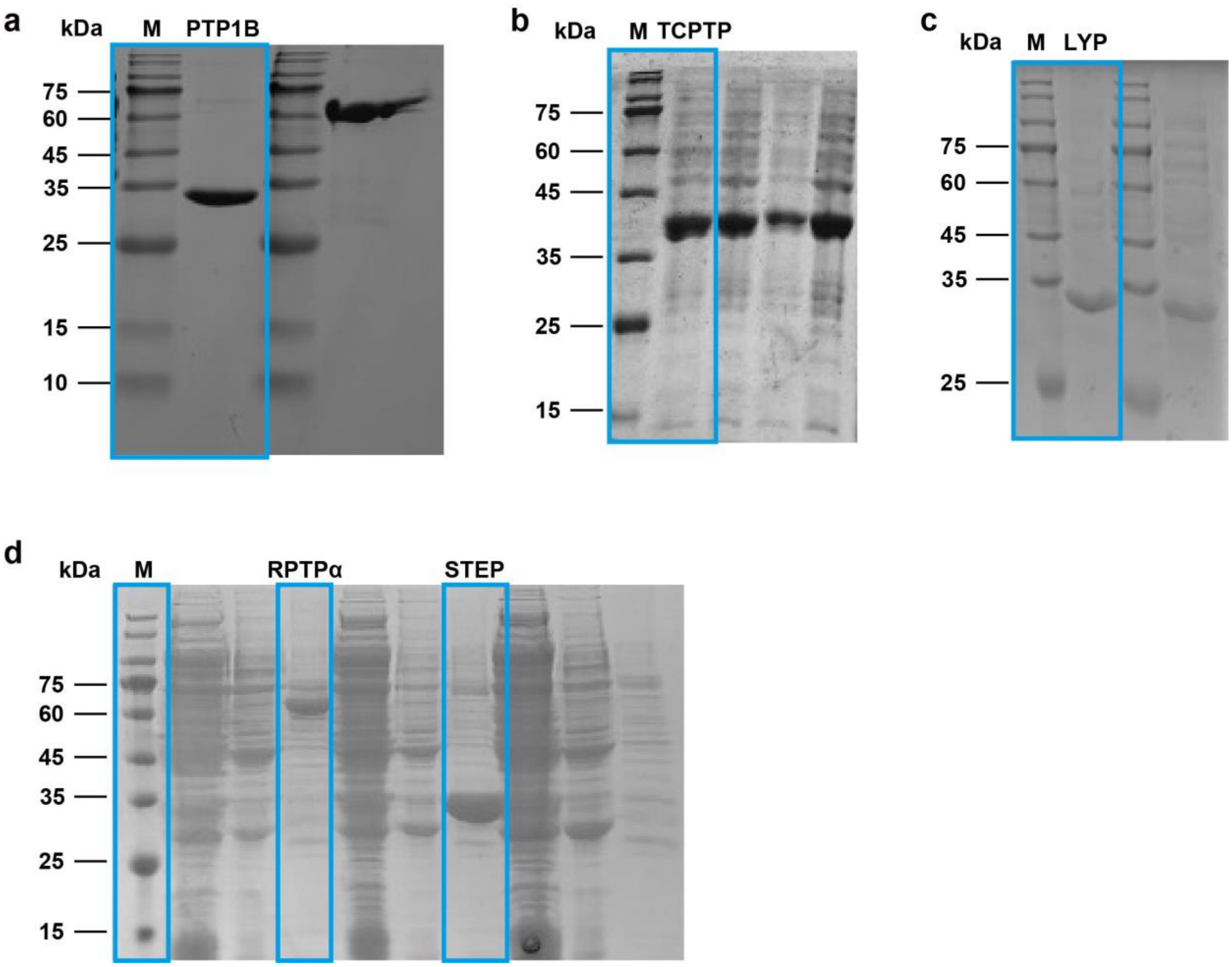

Supplementary Figure 11.

Uncropped blots for Supplementary Figure 2b (a), Supplementary Figure 8a (b), Supplementary Figure 8b (c), and Supplementary Figure 8c (d). Areas enclosed by blue lines are cropped.

Supplementary Table 1. **Primers for PTP1B mutations.**

Name	Sequence (5'⇨3')
PTP1B_N193A_F	AGCCTCATTCTTG**GCC**TTTCTTTTCAAAG
PTP1B_N193A_R	CTTTGAAAAGAAA**GGC**CAAGAATGAGGCT
PTP1B_F196A_F	GAACTTTCTT**GCC**AAAGTCCGAGAGTCAGG
PTP1B_F196A_R	CCTGACTCTCGGACTTT**GGC**AAGAAAGTTC
PTP1B_F196R_F	GAACTTTCTT**CGC**AAAGTCCGAGAGTCAGG
PTP1B_F196R_R	CCTGACTCTCGGACTTT**GCG**AAGAAAGTTC
PTP1B_E276A_F	CTGGCTGTGATC**GCC**GGTGCCAAAT
PTP1B_E276A_R	ATTTGGCACC**GGC**GATCACAGCCAG
PTP1B_W291A_F	CGTGCAGGATCAG**GCC**AAGGAGCTTTCCCA
PTP1B_W291A_R	TGGGAAAGCTCCTT**GGC**CTGATCCTGCACG
