# Supplementary material for: Structural basis of Fumosorinone-mediated allosteric inhibition of PTP1B for cancer immunotherapy
Source: Commun Biol. 2026 May 28;9:729. doi: 10.1038/s42003-026-10329-2 (PMC13219501; doi:10.1038/s42003-026-10329-2)
Supplement: Supplementary file 3 — Description of additional supplementary word file [file 42003_2026_10329_MOESM3_ESM.pdf]

Description of additional supplementary word file

Supplementary Data 1: Contains the input structure file used for molecular dynamics (MD) simulations described in this study.

Supplementary Data 2: Provides the 100-ns MD simulation output files corresponding to the results reported in the main text.

Supplementary Data 3: Figure 1e, Supplementary Figure 1f, Supplementary Figure 2a are list in Supplementary Data 3.

Supplementary Data 4: The source data of crystals, PDB and MTZ files are concluded in Supplementary Data 4.
