# Supplementary material for: Structural basis of Fumosorinone-mediated allosteric inhibition of PTP1B for cancer immunotherapy
Source: Commun Biol. 2026 May 28;9:729. doi: 10.1038/s42003-026-10329-2 (PMC13219501; doi:10.1038/s42003-026-10329-2)
Supplement: Supplementary file 8 — nr-reporting-summary [file 42003_2026_10329_MOESM8_ESM.pdf]

nature portfolio

Corresponding author(s): Zhiguang Yuchi

Last updated by author(s): May 9, 2026

Reporting Summary

Nature Portfolio wishes to improve the reproducibility of the work that we publish. This form provides structure for consistency and transparency in reporting. For further information on Nature Portfolio policies, see our Editorial Policies and the Editorial Policy Checklist.

Statistics

For all statistical analyses, confirm that the following items are present in the figure legend, table legend, main text, or Methods section.

n/a	Confirmed	
☐	☒	The exact sample size (n) for each experimental group/condition, given as a discrete number and unit of measurement
☐	☒	A statement on whether measurements were taken from distinct samples or whether the same sample was measured repeatedly
☐	☒	The statistical test(s) used AND whether they are one- or two-sided<br>*Only common tests should be described solely by name; describe more complex techniques in the Methods section.*
☒	☐	A description of all covariates tested
☒	☐	A description of any assumptions or corrections, such as tests of normality and adjustment for multiple comparisons
☐	☒	A full description of the statistical parameters including central tendency (e.g. means) or other basic estimates (e.g. regression coefficient) AND variation (e.g. standard deviation) or associated estimates of uncertainty (e.g. confidence intervals)
☒	☐	For null hypothesis testing, the test statistic (e.g. F, t, r) with confidence intervals, effect sizes, degrees of freedom and P value noted<br>*Give P values as exact values whenever suitable.*
☒	☐	For Bayesian analysis, information on the choice of priors and Markov chain Monte Carlo settings
☒	☐	For hierarchical and complex designs, identification of the appropriate level for tests and full reporting of outcomes
☐	☒	Estimates of effect sizes (e.g. Cohen's d, Pearson's r), indicating how they were calculated

Our web collection on statistics for biologists contains articles on many of the points above.

Software and code

Policy information about availability of computer code

Data collection	*Provide a description of all commercial, open source and custom code used to collect the data in this study, specifying the version used OR state that no software was used.*
Data analysis	*Provide a description of all commercial, open source and custom code used to analyse the data in this study, specifying the version used OR state that no software was used.*

For manuscripts utilizing custom algorithms or software that are central to the research but not yet described in published literature, software must be made available to editors and reviewers. We strongly encourage code deposition in a community repository (e.g. GitHub). See the Nature Portfolio guidelines for submitting code & software for further information.

Data

Policy information about availability of data

All manuscripts must include a data availability statement. This statement should provide the following information, where applicable:

- Accession codes, unique identifiers, or web links for publicly available datasets
- A description of any restrictions on data availability
- For clinical datasets or third party data, please ensure that the statement adheres to our policy

rystallographic data for the structures reported in this article have been deposited at the Protein Data Bank, under deposition numbers PDB 9LP5 and 9LOK. All

other relevant data generated and analysed during this study, which include experimental, crystallographic and computational data, are included in this article and its supplementary information.

Research involving human participants, their data, or biological material

Policy information about studies with human participants or human data. See also policy information about sex, gender (identity/presentation), and sexual orientation and race, ethnicity and racism.

Reporting on sex and gender	*Use the terms sex (biological attribute) and gender (shaped by social and cultural circumstances) carefully in order to avoid confusing both terms. Indicate if findings apply to only one sex or gender; describe whether sex and gender were considered in study design; whether sex and/or gender was determined based on self-reporting or assigned and methods used. Provide in the source data disaggregated sex and gender data, where this information has been collected, and if consent has been obtained for sharing of individual-level data; provide overall numbers in this Reporting Summary. Please state if this information has not been collected. Report sex- and gender-based analyses where performed, justify reasons for lack of sex- and gender-based analysis.*
Reporting on race, ethnicity, or other socially relevant groupings	*Please specify the socially constructed or socially relevant categorization variable(s) used in your manuscript and explain why they were used. Please note that such variables should not be used as proxies for other socially constructed/relevant variables (for example, race or ethnicity should not be used as a proxy for socioeconomic status). Provide clear definitions of the relevant terms used, how they were provided (by the participants/respondents, the researchers, or third parties), and the method(s) used to classify people into the different categories (e.g. self-report, census or administrative data, social media data, etc.) Please provide details about how you controlled for confounding variables in your analyses.*
Population characteristics	*Describe the covariate-relevant population characteristics of the human research participants (e.g. age, genotypic information, past and current diagnosis and treatment categories). If you filled out the behavioural & social sciences study design questions and have nothing to add here, write "See above."*
Recruitment	*Describe how participants were recruited. Outline any potential self-selection bias or other biases that may be present and how these are likely to impact results.*
Ethics oversight	*Identify the organization(s) that approved the study protocol.*

Note that full information on the approval of the study protocol must also be provided in the manuscript.

Field-specific reporting

Please select the one below that is the best fit for your research. If you are not sure, read the appropriate sections before making your selection.

☒ Life sciences ☐ Behavioural & social sciences ☐ Ecological, evolutionary & environmental sciences

For a reference copy of the document with all sections, see nature.com/documents/nr-reporting-summary-flat.pdf

Life sciences study design

All studies must disclose on these points even when the disclosure is negative.

Sample size	The sample sizes were chosen based on commonly accepted standards in the field and our previous studies, which demonstrated sufficient statistical power to detect biologically meaningful differences. For in vitro assays, each condition was performed with three independent biological replicates. For in vivo experiments, six animals per group were used, which provides adequate power to identify statistically significant differences while minimizing animal use in accordance with ethical guidelines.
Data exclusions	No data were excluded from the analyses.
Replication	To verify the reproducibility of our experimental findings, all key experiments—including biochemical assays, gene expression analyses, and functional validations—were independently repeated at least three times under identical conditions. Consistent results were obtained across all replicates, confirming the reliability of our observations. Data variability was minimal and within expected biological ranges.
Randomization	This study did not involve allocation of samples into experimental groups. All samples were collected and processed according to predefined experimental conditions without group randomization. Therefore, random allocation and covariate control are not relevant to this study.
Blinding	The investigators were not blinded to group allocation during data collection and analysis. Blinding was not applicable to this study because the experimental groups were defined by distinct treatment conditions (control vs. compound-treated) that produced clear and measurable molecular and phenotypic differences.

Reporting for specific materials, systems and methods

We require information from authors about some types of materials, experimental systems and methods used in many studies. Here, indicate whether each material, system or method listed is relevant to your study. If you are not sure if a list item applies to your research, read the appropriate section before selecting a response.

Materials & experimental systems

n/a	Involved in the study
☐	☒ Antibodies
☐	☒ Eukaryotic cell lines
☒	☐ Palaeontology and archaeology
☐	☒ Animals and other organisms
☒	☐ Clinical data
☒	☐ Dual use research of concern
☒	☐ Plants

Methods

n/a	Involved in the study
☒	☐ ChIP-seq
☐	☒ Flow cytometry
☒	☐ MRI-based neuroimaging

Antibodies

Antibodies used	CD45 (103108, 103126), CD3 (100218), CD4 (100428), CD8 (100752), CD25 (102036), CD69 (104512), NKp46 (137618), CD19 (152406), CD11b (101224, 101263,), Ly6C (128033), Ly6G (127639), F4/80 (123114), CD206 (141732), iNOS (696806), Gr-1 (108428), CD11c (117334), MHC-II (107614), PD-1 (135228), GZMB (372208) and Foxp3 (126403) (all from BioLegend)
Validation	Each primary antibody used in this study was validated for its specificity and application in the corresponding species. The validation information was reviewed from the manufacturer's technical datasheet, relevant research citations, and antibody databases such as UniProt.

Eukaryotic cell lines

Policy information about cell lines and Sex and Gender in Research

Cell line source(s)	The mouse colon adenocarcinoma MC38 cell line and the mouse bladder cancer MB49 cell line were both obtained from the Shanghai Cell Bank, Chinese Academy of Sciences.
Authentication	All cell lines used in this study were obtained from the Cell Bank of the Chinese Academy of Sciences (Shanghai, China). According to the supplier's documentation, all cell lines were authenticated by short tandem repeat (STR) profiling and tested to be free of mycoplasma contamination. No additional authentication was performed by the authors.
Mycoplasma contamination	The cell lines were tested to be free of mycoplasma contamination.
Commonly misidentified lines (See ICLAC register)	*Name any commonly misidentified cell lines used in the study and provide a rationale for their use.*

Animals and other research organisms

Policy information about studies involving animals; ARRIVE guidelines recommended for reporting animal research, and Sex and Gender in Research

Laboratory animals	Female C57BL/6J mice (6-8 weeks)
Wild animals	This study did not involve the observation or capture of wild animals. All experiments were performed using laboratory animals maintained under controlled conditions in accordance with institutional and national guidelines for the care and use of laboratory animals.
Reporting on sex	All experiments in this study were conducted using female mice. The choice of sex was based on consistency with previous research in the field and to minimize variability associated with hormonal cycles in mixed-sex cohorts. Sex was therefore not considered as an independent experimental variable, and no sex-based analysis was performed. No male animals were included.
Field-collected samples	The study did not involve samples collected from the field.
Ethics oversight	All animal experiments were conducted in accordance with the guidelines of the Nankai University Research Institute Research Ethics Committee (protocol registration number: A-2018-0306).

Note that full information on the approval of the study protocol must also be provided in the manuscript.

Plants

Seed stocks	*Report on the source of all seed stocks or other plant material used. If applicable, state the seed stock centre and catalogue number. If plant specimens were collected from the field, describe the collection location, date and sampling procedures.*
Novel plant genotypes	*Describe the methods by which all novel plant genotypes were produced. This includes those generated by transgenic approaches, gene editing, chemical/radiation-based mutagenesis and hybridization. For transgenic lines, describe the transformation method, the number of independent lines analyzed and the generation upon which experiments were performed. For gene-edited lines, describe the editor used, the endogenous sequence targeted for editing, the targeting guide RNA sequence (if applicable) and how the editor was applied.*
Authentication	*Describe any authentication procedures for each seed stock used or novel genotype generated. Describe any experiments used to assess the effect of a mutation and, where applicable, how potential secondary effects (e.g. second site T-DNA insertions, mosiacism, off-target gene editing) were examined.*

Flow Cytometry

Plots

Confirm that:

☒ The axis labels state the marker and fluorochrome used (e.g. CD4-FITC).

☒ The axis scales are clearly visible. Include numbers along axes only for bottom left plot of group (a 'group' is an analysis of identical markers).

☒ All plots are contour plots with outliers or pseudocolor plots.

☒ A numerical value for number of cells or percentage (with statistics) is provided.

Methodology

Sample preparation	Tumor tissues were enzymatically dissociated into single-cell suspensions using a digestion solution containing Collagenase IV (1 mg/mL), Hyaluronidase (1 mg/mL), and DNase I (20 U/mL) (Sigma). The isolated cells were stained with a viability dye at room temperature for 15 min, followed by incubation with mouse anti-CD16/CD32 antibodies for 15 min. Subsequently, fluorescence-conjugated antibodies were applied for 30 min to label cell-surface markers, including CD45 (103108, 103126), CD3 (100218), CD4 (100428), CD8 (100752), CD25 (102036), CD69 (104512), NKp46 (137618), CD19 (152406), CD11b (101224, 101263,), Ly6C (128033), Ly6G (127639), F4/80 (123114), CD206 (141732), iNOS (696806), Gr-1 (108428), CD11c (117334), MHC-II (107614), PD-1 (135228) (all from BioLegend). After washing with PBS, cells were fixed, permeabilized, and subjected to intracellular staining for 30 min with GZMB (372208) and Foxp3 (126403) (BioLegend). Flow cytometric analysis was then performed.
Instrument	BD FACSCalibur™ Flow Cytometer
Software	Flowjo, Graphpad
Cell population abundance	Following cell sorting, the abundance and purity of the relevant populations were assessed by flow cytometry. Post-sort fractions were re-analyzed using the same gating strategy applied during sorting to confirm population identity. The purity of each sorted population exceeded 95%, as determined by the proportion of target marker-positive cells within the gated population.
Gating strategy	The gating strategy for flow cytometry data of T cell function and immune cell infiltration proportions in the tumor immune microenvironment is as follows: first, gate the main cell population, and exclude doublets or multiplets using FSC-H vs. FSC-A to ensure single-cell analysis. Live cells are selected using a viability dye, excluding dead cells. CD45 is used to gate tumor-infiltrating immune cells (CD45+). From the CD45+ cells, CD3+ T cells are gated. CD8+ cytotoxic T cells and CD4+ helper T cells are further distinguished. Under this gate, additional markers are gated to analyze the functional status of T cells in the tumor microenvironment, such as cytotoxicity (GZMB), activation (IFN-γ), exhaustion (PD-1), and Tregs (Foxp3), among others.

☒ Tick this box to confirm that a figure exemplifying the gating strategy is provided in the Supplementary Information.
